# Generalized joint hypermobility and functional recovery after THA for unilateral Hartofilakidis type C developmental dysplasia of the hip: a retrospective cohort study

**DOI:** 10.1186/s42836-026-00405-7

**Published:** 2026-06-09

**Authors:** Yuhang Wang, Zhi Li, Xingshi Yuan, Yuxin Xia, Ming Jiao, Guoyuan Li, Xifu Shang

**Affiliations:** 1 Department of Orthopedics, The First Affiliated Hospital of USTC, Hefei, 230001 Anhui China; 2 Division of Life Sciences and Medicine, University of Science and Technology of China, Hefei, 230026 Anhui China

## Abstract

**Background:**

Generalized joint hypermobility (GJH) is common in developmental dysplasia of the hip (DDH) and may affect recovery after total hip arthroplasty (THA) for Hartofilakidis type C, but its impact on the trajectory of postoperative functional recovery in this severe subgroup has not been clearly characterized.

**Methods:**

We retrospectively reviewed patients aged 18–40 years who underwent THA for unilateral Hartofilakidis type C DDH (2018–2023). GJH was defined as a Beighton score ≥ 5. All procedures used a direct anterior approach with proximal femoral osteotomy for exposure and selective femoral shortening at the lesser trochanter level when reduction would otherwise impose excessive neurovascular tension or unacceptable residual leg-length discrepancy (LLD). Radiographic parameters and LLD were assessed. Harris Hip Score (HHS) was recorded preoperatively and at 1, 3, 6, and 12 months. Longitudinal HHS was analysed using a linear mixed-effects model; effect sizes were quantified by Cohen’s d and partial η^2^.

**Results:**

Fifty-eight patients were included (21 GJH, 37 non-GJH). Postoperative LLD was reduced without a between-group difference. Cup position at 6 months was similar; femoral offset tended to be higher in GJH (*p* = 0.057). Femoral shortening distance was smaller in GJH (*p* < 0.001). Observed HHS was lower in GJH at 1–6 months (*p* < 0.001) but similar at 12 months. The linear mixed-effects model (LMM) showed significant time, group, and time × group effects (*p* < 0.001). Adjusted between-group differences were largest at 1 month and progressively diminished, becoming non-significant at 12 months. One dislocation occurred in the GJH group; no revisions were required.

**Conclusions:**

In young adults with unilateral Hartofilakidis type C DDH, GJH did not compromise 12-month hip function after THA but was associated with a slower early recovery trajectory, with the largest deficit during the first 1–3 postoperative months.

**Supplementary Information:**

The online version contains supplementary material available at 10.1186/s42836-026-00405-7.

## Introduction

Developmental dysplasia of the hip (DDH) is a congenital disorder characterized by structural abnormalities of the acetabulum, femoral head, and/or proximal femur [1]. Severe DDH often causes chronic pain, major functional limitation, and reduced quality of life [2]. Hartofilakidis Type C (high dislocation) represents the most severe form and remains technically demanding to treat [3, 4].

Generalized joint hypermobility (GJH) is the most extensive manifestation of joint hypermobility. In GJH, generalized connective-tissue laxity permits joint motion beyond normal limits and may contribute to recurrent instability and chronic, widespread musculoskeletal pain [5–7]. GJH is conventionally screened with the Beighton score, a nine-item instrument with age-stratified thresholds (≥ 6 for prepubertal children, ≥ 5 for postpubertal individuals up to age 50, and ≥ 4 for those over 50) [8]. Prior studies have reported that GJH occurs in approximately 27% of patients with DDH and in up to 77.9% of DDH patients without established osteoarthritis [9, 10]. These findings suggest that GJH is clinically relevant in DDH and may be especially important in patients with severe deformity.

Unilateral Hartofilakidis Type C DDH is often associated with substantial leg-length discrepancy (LLD), severe limp, gait abnormalities, and functional limitation. LLD and altered mechanics have been associated with secondary symptoms such as low back pain and compensatory spinal curvature [11]. In young patients, particularly women, marked limb shortening and gait impairment can be indications for surgical reconstruction even when radiographic osteoarthritis is not advanced [12]. In such cases, reconstruction is often pursued to restore limb length, improve gait, and relieve symptoms. Total hip arthroplasty (THA) is an established treatment for Hartofilakidis Type C DDH [13, 14]. However, concomitant GJH may introduce additional challenges. Soft-tissue laxity can complicate intraoperative stability management and may influence restoration of offset and leg length. It may also increase the risk of instability-related complications, such as dislocation [15], and could affect the pace of postoperative functional recovery.

Therefore, this study aimed to determine whether GJH is associated with longitudinal postoperative Harris Hip Score (HHS) recovery after THA in patients with Hartofilakidis Type C DDH. We also characterized baseline radiographic morphology and perioperative reconstruction parameters by GJH status. Finally, we examined prespecified clinical and radiographic factors associated with postoperative recovery.

## Materials and methods

### Patient enrollment

This retrospective study used data from the Orthopedic Department of the First Affiliated Hospital of the University of Science and Technology of China. The institutional review board approved the study (No. 2024-RE-436). Written informed consent was obtained from all participants.

GJH was assessed using the Beighton score. The score ranges from 0 to 9 and includes bilateral fifth metacarpophalangeal joints, thumbs, elbows, and knees, as well as trunk forward flexion. A Beighton score ≥ 5 was used to define GJH. The detailed criteria are provided in Supplementary File 1. All patients were evaluated on admission by trained ward nurses, and the Beighton score was recorded in the medical record.

Patients were included if they: (1) underwent THA between January 1, 2018, and January 31, 2023; (2) were 18–40 years old at the time of surgery; and (3) had Hartofilakidis type C DDH confirmed on standardized anteroposterior pelvic radiographs.

Patients were excluded if they: (1) had bilateral Hartofilakidis type C DDH; (2) had other hip disorders; (3) had inflammatory arthropathy (e.g., rheumatoid arthritis) or other connective tissue/systemic disorders associated with joint laxity (e.g., Marfan syndrome or neuromuscular disease); (4) had prior hip trauma or prior hip surgery; (5) had no preoperative Beighton score documented; or (6) had incomplete follow-up data.

**Fig. 1:**
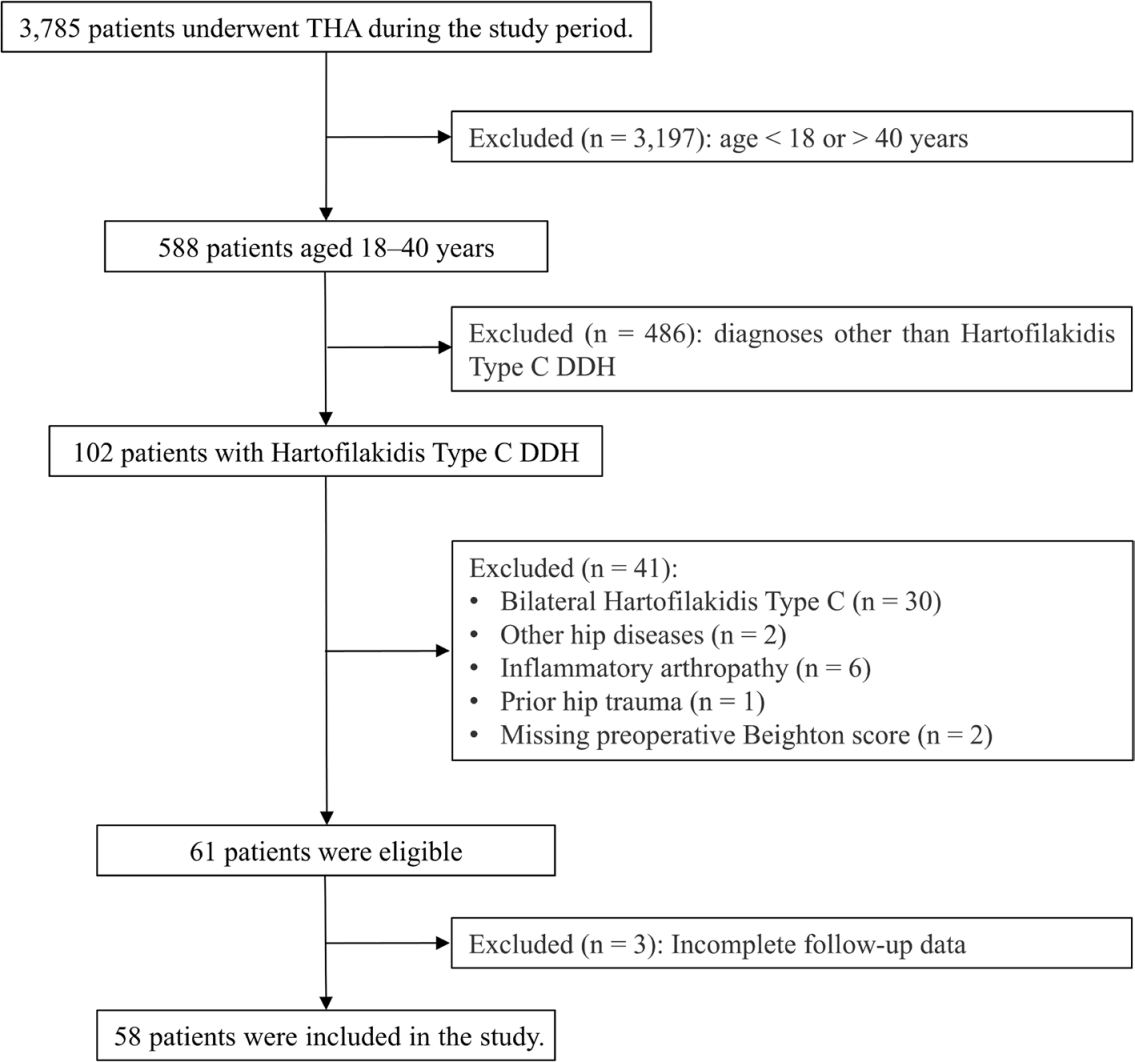
Patient selection flowchart

All eligible patients within the study period were screened consecutively, and no case was excluded selectively based on outcome. Between January 2018 and January 2023, a total of 3,785 patients underwent THA at our institution. After applying the age criterion (18–40 years), 588 patients remained, of whom 102 were diagnosed with Hartofilakidis type C DDH. The exclusion criteria removed bilateral high dislocation (*n* = 30), other hip disorders (*n* = 2), inflammatory arthropathy (*n* = 6), prior hip trauma (*n* = 1), missing preoperative Beighton score (*n* = 2), and incomplete follow-up (*n* = 3), yielding the final analytic cohort of 58 patients (Fig. 1).

### Operative technique

All procedures were performed by a single surgical team using the direct anterior approach (DAA). All patients received a PINNACLE acetabular cup (DePuy Synthes) and a hydroxyapatite-coated tapered femoral stem (DePuy Synthes). A capsulotomy was performed to expose the femoral head and acetabulum. The deformed femoral head was excised in situ. Additional soft-tissue releases were performed when needed. A cementless acetabular component was press-fit into the prepared acetabulum. Supplemental screws were used when initial cup stability was inadequate.

After acetabular preparation, a proximal femoral osteotomy (PFO) for exposure was performed. The osteotomy started at the proximal border of the lesser trochanter and extended obliquely distally and laterally. The lesser trochanter remained attached to the femoral shaft, which stayed in continuity. No femoral segment was resected during this step. The purpose was to improve exposure, facilitate canal preparation, and allow controlled reduction.

Femoral anteversion was determined with reference to the medial epicondyle and the patella. Before broaching, a prophylactic cerclage wire was placed around the femoral shaft just distal to the osteotomy site to prevent iatrogenic fracture during canal preparation. The medullary canal was then broached through the distal cut surface.

A secondary femoral shortening osteotomy, performed at the level of the lesser trochanter, was used selectively when reduction would otherwise cause excessive neurovascular tension or unacceptable LLD. The femoral osteotomy shortening distance (F-OSD) was recorded.

After femoral broaching, the final broach was left in place, and a trial neck was used to assess leg length. Using an oscillating saw, a longitudinal bone groove was prepared on the inner cortical surface of the proximal fragment to match the cross-sectional contour of the proximal stem. The groove width matched the thickness of the selected stem trial, and the groove depth served two purposes: (a) it accommodated the proximal portion of the stem so that the fragment seated firmly against the shoulder of the prosthesis, and (b) it allowed intraoperative adjustment of femoral offset (FO)—a shallower groove increased offset by translating the proximal fragment more laterally, whereas a deeper groove decreased offset. The proximal fragment was reduced and temporarily held with reduction forceps to assess stability.

After implantation of the definitive stem, the proximal fragment was reduced onto the stem to recreate a circumferential cortical envelope around its proximal portion, and cerclage wires were used to reattach the fragment to the lateral femoral shaft. Autograft harvested from the femoral head was packed into the osteotomy gap to promote healing. Details and mid-term reliability of this proximal femoral reconstruction technique have been described previously [16, 17].

### Postoperative rehabilitation and follow-up

All patients followed a standardized, stage-based rehabilitation protocol that was uniform across groups and was not stratified by GJH status. The protocol comprised four phases: (a) acute phase (weeks 1–2): pain control, prevention of thromboembolic and wound complications, and basic functional mobilization under standard hip precautions; ambulation with crutches was permitted from postoperative day 2, and the operated limb was kept non–weight-bearing during this period; (b) early recovery (weeks 3–4): progression from non–weight-bearing to partial weight-bearing, with initiation of active hip and knee range-of-motion exercises; (c) intermediate recovery (weeks 5–12): balance and proprioceptive training, progressive transition to full weight-bearing, and correction of pelvic obliquity; (d) late recovery (weeks 13–24): progressive resistance training of the hip musculature (particularly the abductors), gait retraining, and endurance training. Adherence was reinforced through preoperative education, in-hospital supervision, scheduled outpatient review, and structured telephone follow-up.

**Fig. 2:**
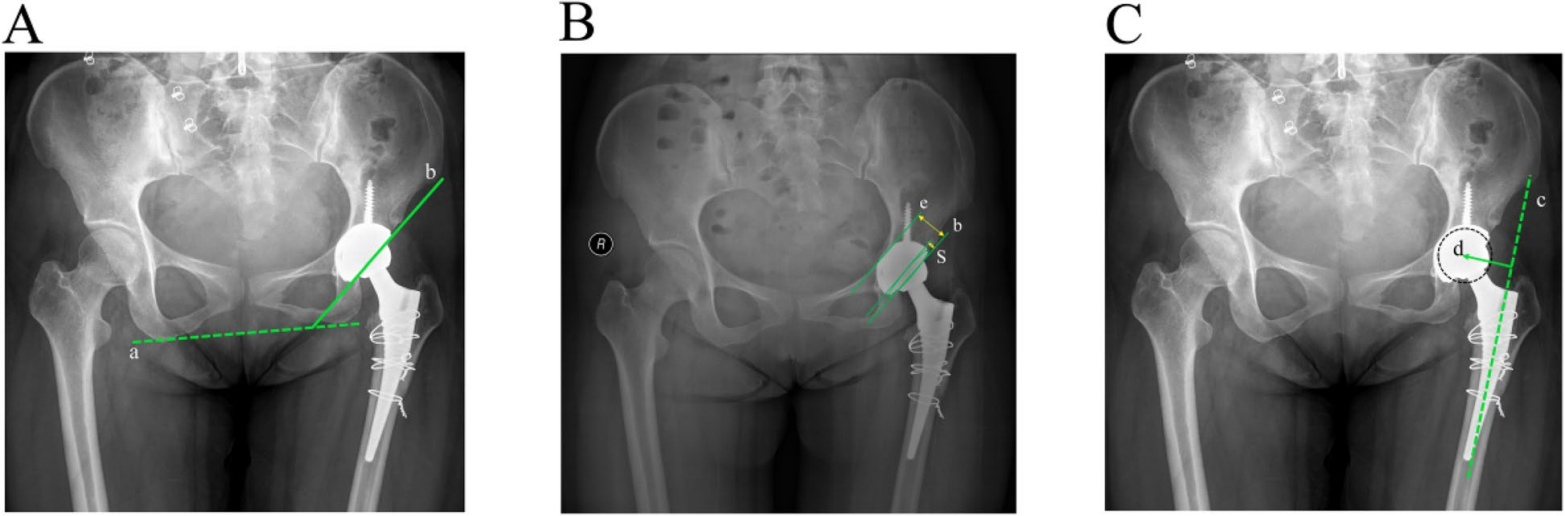
Measurement of prosthesis position on radiographs. Reference points and lines: (a) line through the ischial tuberosities; (b) line connecting the inner and outer edges of the acetabular cup; (c) longitudinal axis of the femoral stem; (d) centre of the prosthetic femoral head; (e) medial apex of the acetabular cup. **A** Acetabular inclination angle (AIA) is defined as the angle between line (b) and line (a). **B** Acetabular anteversion angle (AVA), where S is the transverse diameter of the cup ellipse, and eb is the distance from the outer rim of the acetabular cup to the medial apex; AVA was calculated as: AVA = 48.05 × (S/eb) − 0.3. **C** Femoral offset (FO) is defined as the perpendicular distance from point (d) to line (c)

Follow-up included radiographic and clinical assessments. Preoperative imaging evaluation included the Tönnis grade of osteoarthritis, lumbar Cobb angle, and lumbar lordosis (LL). LLD was assessed clinically with the patient in the supine position. True limb length was measured as the distance from the anterior superior iliac spine to the ipsilateral medial malleolus using a tape measure on both sides. Postoperative LLD was reassessed using the same protocol to quantify limb-length change. At 6 months postoperatively, implant position was assessed on radiographs by measuring the acetabular inclination angle (AIA), acetabular anteversion angle (AVA), and femoral offset (FO) (Fig. 2).

Clinical outcomes were assessed using the Harris Hip Score (HHS). The HHS was recorded preoperatively and at 1, 3, 6, and 12 months postoperatively. Thereafter, the HHS was collected annually until the final follow-up. Baseline demographic data were recorded for all patients. Major postoperative complications were documented, including infection, nerve injury, prosthetic dislocation, nonunion at the osteotomy site, and periprosthetic fracture.

### Linear mixed-effects model for HHS

Hip function was assessed using the Harris Hip Score (HHS). A linear mixed-effects model (LMM) was fitted with HHS as the dependent variable. Time (preoperative and 1, 3, 6, and 12 months postoperatively) was modelled as a categorical fixed effect, with the preoperative assessment as the reference level. Fixed effects included time, group (GJH vs non-GJH), and the time × group interaction. Candidate covariates were identified using univariable between-group analyses of baseline and radiographic variables. Variables with *p* < 0.10 were entered into the multivariable LMM. Patient ID was included as a random intercept to account for within-subject correlation. Repeated-measures covariance structures were compared using Akaike and Bayesian information criteria and model convergence. A heterogeneous first-order autoregressive structure [ARH(1)] was selected. Model parameters were estimated using restricted maximum likelihood (REML).

To assess the robustness of the principal findings to the baseline imbalance in Tönnis grade, a prespecified sensitivity analysis was performed in patients with Tönnis grade 0–1 (*n* = 47), refitting the LMM with the same fixed-effect, random-effect, and covariance structures (Supplementary Table S1). To complement statistical inference, effect sizes were quantified as Cohen’s d with 95% CIs for between-group HHS comparisons at each time point, and partial η^2^ was computed for each fixed effect in the LMM.

### Statistical analysis

Descriptive analyses and between-group comparisons were performed in R (version 4.3.0). Normality of continuous variables was assessed using the Shapiro–Wilk test. Normally distributed variables are presented as mean ± standard deviation (SD). Non-normally distributed variables are presented as median (interquartile range, IQR). Categorical variables are presented as a number (%). Between-group comparisons used the chi-square test or Fisher’s exact test, as appropriate.

Longitudinal HHS was analysed using the LMM implemented in SPSS Statistics (version 26.0; IBM Corp., Armonk, NY, USA). We report Type III tests of fixed effects, fixed-effect estimates with 95% confidence intervals, and covariance parameter estimates. Estimated marginal means (EMMs) were obtained from the model. Between-group contrasts were performed at each time point. P values for pairwise contrasts were adjusted using the Bonferroni method. Missing follow-up data were handled within the LMM framework using all available observations. REML estimation accommodates incomplete longitudinal data under the missing-at-random assumption. All tests were two-sided, and statistical significance was set at *p* < 0.05.

## Results

### Patient demographics

A total of 58 patients were included, with 21 in the GJH group and 37 in the non-GJH group. The mean age of the analytic cohort was 30.16 ± 5.92 years, and the mean follow-up duration across both groups was 51.05 ± 16.66 months. Age was comparable between groups (*p* = 0.472). Length of hospital stay did not differ between groups (*p* = 0.866). Follow-up duration was similar (*p* = 0.495). As expected, the Beighton score was higher in the GJH group (*p* < 0.001). Sex distribution, BMI category, education level, and urban/rural residence were comparable between groups (*p* > 0.05). The distribution of Tönnis grades differed between groups (*p* = 0.038). The GJH group consisted mainly of Tönnis grade 0–1 (grade 0, 71.43%; grade 1, 28.57%) and had no cases of grade 2–3. The non-GJH group had a higher proportion of grade 2–3 (grade 2, 24.32%; grade 3, 5.41%). Baseline demographic characteristics are presented in Table 1.

**Table 1:** Basic information for patients with Hartofilakidis type C DDH

Variables	Total (*n* = 58)	GJH (*n* = 21)	non-GJH (*n* = 37)	Statistic	*p*
Age	30.16 ± 5.92	30.90 ± 5.48	29.73 ± 6.19	*t* = 0.72	0.472^a^
SEX, *n* (%)				χ^2^ = 1.76	0.184^b^
Male	9 (15.52)	1 (4.76)	8 (21.62)		
Female	49 (84.48)	20 (95.24)	29 (78.38)		
BMI, *n* (%)				χ^2^ = 2.00	0.412^b^
< 18.5	5 (8.62)	3 (14.29)	2 (5.41)		
18.5–24.9	44 (75.86)	14 (66.67)	30 (81.08)		
> = 25	9 (15.52)	4 (19.05)	5 (13.51)		
Education, *n* (%)				χ^2^ = 3.19	0.185^b^
Primary education	2 (3.45)	2 (9.52)	0 (0.00)		
Secondary education	25 (43.10)	9 (42.86)	16 (43.24)		
Tertiary education	31 (53.45)	10 (47.62)	21 (56.76)		
Urban/Rural Classification, *n* (%)				χ^2^ = 0.18	0.671^b^
Urban	27 (46.55)	9 (42.86)	18 (48.65)		
Rural	31 (53.45)	12 (57.14)	19 (51.35)		
Tönnis type, *n* (%)				χ^2^ = 7.87	0.038^b^
0	34 (58.62)	15 (71.43)	19 (51.35)		
1	13 (22.41)	6 (28.57)	7 (18.92)		
2	9 (15.52)	0 (0.00)	9 (24.32)		
3	2 (3.45)	0 (0.00)	2 (5.41)		
Beighton score	3.43 ± 2.71	6.76 ± 0.94	1.54 ± 0.99	*t* = 19.64	< 0.001^a^
LOS (days)	8.64 ± 2.23	8.57 ± 1.66	8.68 ± 2.52	*t* = − 0.17	0.866^a^
Follow-up time (months)	51.05 ± 16.66	49.05 ± 13.24	52.19 ± 18.39	*t* = − 0.69	0.495^a^

The values are given as the mean and the standard deviation

^a^Student’s t-test; ^b^chi-square test

GJH: Generalized Joint Hypermobility; BMI: Body Mass Index; LOS: Length of Stay

### Surgical procedure and radiographic data

Preoperative radiographic parameters differed in lumbar alignment. The GJH group had a larger lumbar Cobb angle than the non-GJH group (*p* = 0.022). LL was similar between groups (*p* = 0.150). Preoperative LLD was comparable (*p* = 0.919). Postoperative LLD was reduced and did not differ significantly between groups (*p* = 0.250).

At 6 months postoperatively, cup position was similar between groups. The AIA did not differ (*p* = 0.873). Acetabular AVA was also comparable (*p* = 0.154). FO tended to be higher in the GJH group, but this did not reach statistical significance (*p* = 0.057).

**Fig. 3:**
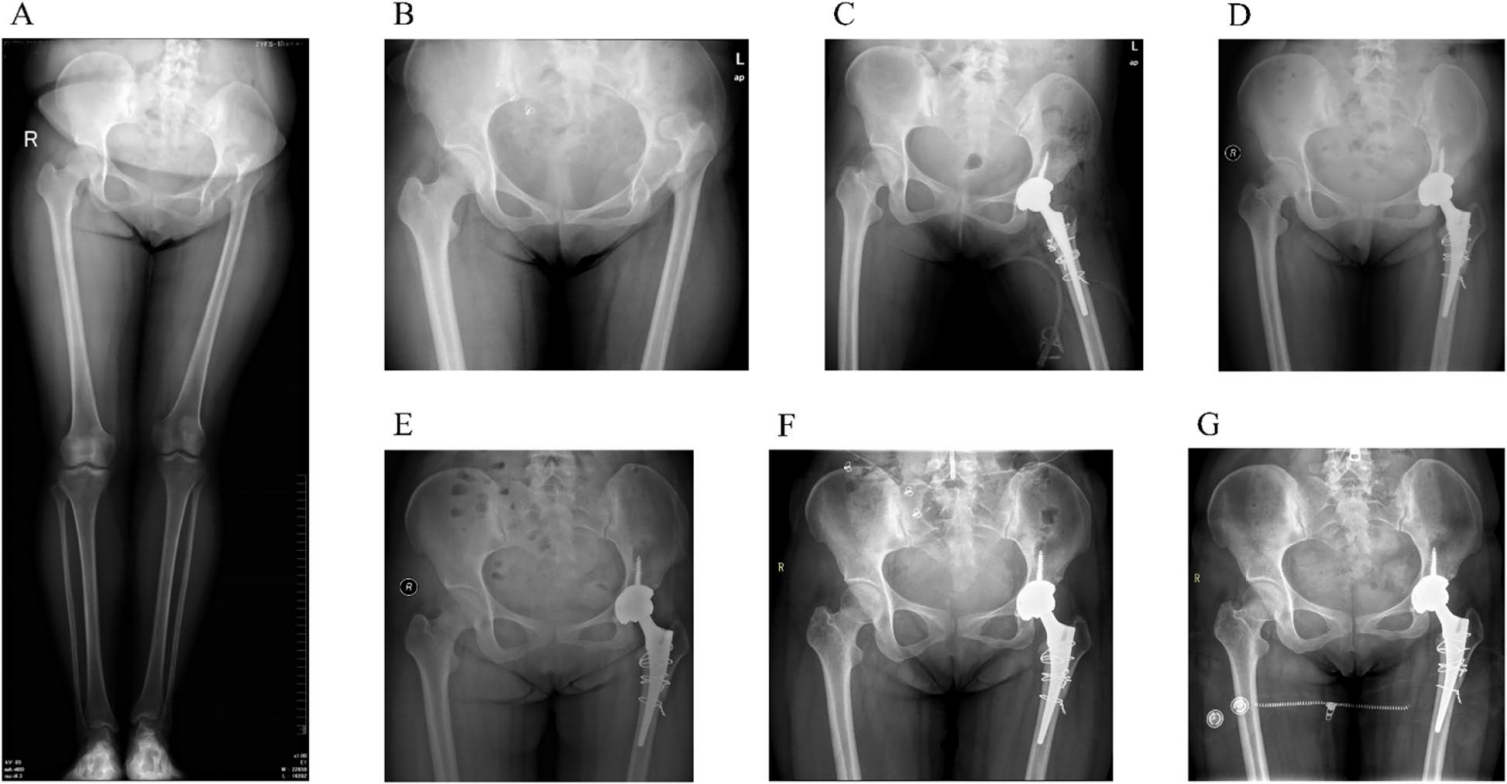
Serial radiographs of a 28-year-old woman with left-sided Hartofilakidis type C developmental dysplasia of the hip before and after total hip arthroplasty. **A** Preoperative full-length standing radiograph showing left high hip dislocation with a leg-length discrepancy of 3.2 cm. **B** Preoperative anteroposterior pelvic radiograph demonstrating the dysplastic acetabulum and proximally migrated femoral head. **C** Anteroposterior pelvic radiograph on postoperative day 1 showing the acetabular component placed at the anatomical hip centre. **D** Anteroposterior pelvic radiograph at 3 months postoperatively showing progressive consolidation at the femoral osteotomy site. **E** Anteroposterior pelvic radiograph at 6 months postoperatively showing complete osteotomy union (acetabular inclination 41.2°, acetabular anteversion 19°, femoral offset 3.21 cm, residual leg-length discrepancy 0.44 cm). **F** and **G** Anteroposterior pelvic radiographs at 12 and 24 months postoperatively, respectively, demonstrating stable implant fixation without evidence of stem subsidence, component loosening, or periprosthetic osteolysis

Among reconstruction-related parameters, the F-OSD was smaller in the GJH group (*p* < 0.001). Other operative parameters (cup size, head size, and stem parameter) were similar between groups (Table 2). A representative case illustrating the surgical reconstruction and serial radiographic course is shown in Fig. 3.

**Table 2:** Comparison of operative parameters and implant characteristics between the GJH and non-GJH groups

Variables	Total (*n* = 58)	GJH (*n* = 21)	non-GJH (*n* = 37)	Statistic	*p*
Cobb (°)	10.08 ± 6.63	12.71 ± 7.11	8.59 ± 5.94	*t* = 2.36	0.022^a^
LL (°)	50.66 ± 16.36	54.74 ± 17.31	48.22 ± 15.50	*t* = 1.46	0.150^a^
LLD (cm)					
Preoperative	3.37 ± 1.08	3.35 ± 1.17	3.38 ± 1.03	*t* = − 0.10	0.919^a^
postoperative	0.97 ± 1.23	1.22 ± 1.78	0.83 ± 0.75	*t* = 1.16	0.250^a^
F-OSD (cm)	1.11 ± 0.69	0.76 ± 0.37	1.30 ± 0.75	*t* = − 3.68	< 0.001^a^
AIA (°)	42.92 ± 6.10	43.10 ± 4.98	42.82 ± 6.72	*t* = 0.16	0.873^a^
AVA (°)	12.52 ± 3.76	13.46 ± 3.32	11.99 ± 3.93	*t* = 1.44	0.154^a^
Offset (cm)	3.39 ± 0.59	3.58 ± 0.52	3.28 ± 0.60	*t* = 1.95	0.057^a^
AC Size (mm)	44.38 ± 1.15	44.38 ± 1.36	44.38 ± 1.04	*t* = 0.01	0.994^a^
PFH Size (mm)	30.21 ± 2.14	30.10 ± 2.41	30.27 ± 2.01	*t* = − 0.30	0.768^a^

The values are given as the mean and the standard deviation

^a^ Student’s t-test

GJH: Generalized Joint Hypermobility; Cobb: Cobb Angle; LL: Lumbar Lordosis; LLD: Limb Length Discrepancy; F-OSD: Femoral Osteotomy Shortening Distance; AIA: Acetabular Inclination Angle; AVA: Acetabular Anteversion Angle; AC Size: Acetabular Cup; PFH Size: Prosthetic Femoral Head Size

### Clinical evaluation

Clinical outcomes were assessed using the Harris Hip Score (HHS). Observed HHS values and between-group effect sizes (Cohen’s d with 95% CIs) are shown in Table 3. Preoperative HHS was similar between groups (*p* = 0.574). Postoperatively, both groups improved. The GJH group had lower observed HHS at 1 month, 3 months, and 6 months (*p* < 0.001). Differences were not significant at 12 months (*p* = 0.455) or at the final follow-up (*p* = 0.728).

**Table 3:** Longitudinal comparison of HHS

Variables	Total (*n* = 58)	GJH (*n* = 21)	non-GJH (*n* = 37)	Statistic	*p*	Cohen’s d	95% CI	
							**Lower Bound**	**Upper Bound**
preoperative	43.10 ± 9.20	42.19 ± 8.12	43.62 ± 9.83	*t* = − 0.57	0.574	− 0.16	− 0.68	0.36
1 month	66.10 ± 6.75	59.52 ± 3.96	69.84 ± 4.88	*t* = − 8.26	< 0.001	− 2.32	− 2.99	− 1.66
3 months	79.29 ± 4.68	75.57 ± 3.34	81.41 ± 3.97	*t* = − 5.68	< 0.001	− 1.59	− 2.18	− 1.00
6 months	84.78 ± 3.20	82.67 ± 2.42	85.98 ± 2.98	*t* = − 4.35	< 0.001	− 1.22	− 1.78	− 0.66
12 months	87.02 ± 2.69	86.71 ± 1.85	87.20 ± 3.07	*t* = − 0.75	0.455	− 0.19	− 0.71	0.32
Final visit	88.15 ± 2.70	88.00 ± 2.00	88.23 ± 3.06	*t* = − 0.35	0.728	− 0.09	− 0.60	0.43

The values are given as the mean and the standard deviation

GJH: Generalized Joint Hypermobility; HHS: Harris Hip Score

Longitudinal HHS was analysed using a multivariable linear mixed-effects model including time, group (GJH vs non-GJH), and the time × group interaction, with adjustment for covariates entered according to the prespecified screening criterion (Tönnis grade, lumbar Cobb angle, FO, and F-OSD). Type III tests showed significant effects of time (*p* < 0.001), group (*p* < 0.001), and time × group (*p* < 0.001). Tönnis grade was not independently associated with longitudinal HHS (*p* = 0.145). Lumbar Cobb angle, FO, and F-OSD were also not significant predictors (*p* > 0.05) (Table 4).

**Table 4:** Significance testing of fixed effects in a linear mixed-effects model

	**Num DF**	**Den DF**	**F**	***p***	**partial η**^**2**^
Intercept	1	51.50	998.97	< 0.001	0.95
Time	4	59.97	510.28	< 0.001	0.97
GJH Status	1	81.72	21.34	< 0.001	0.21
Tönnis Classification	3	49.72	1.88	0.145	0.10
Cobb Angle (°)	1	49.72	0.18	0.671	0.01
Femoral Offset (cm)	1	49.72	2.08	0.156	0.04
F-OSD (cm)	1	49.72	2.75	0.103	0.05
Time × GJH Status	4	59.97	21.58	< 0.001	0.59

Dependent variable: Harris Hip Score (HHS)

Num DF = Numerator degrees of freedom; Den DF = Denominator degrees of freedom; GJH: Generalized Joint Hypermobility; F-OSD: Femoral—Osteotomy Shortening Distance

Model-based estimated marginal means showed lower adjusted HHS in the GJH group at 1 month, 3 months, and 6 months (*p* < 0.001). No between-group difference was observed preoperatively (*p* = 0.462) or at 12 months (*p* = 0.356) (Table 5).

**Table 5:** Adjusted estimated marginal means of HHS by follow-up time and between-group contrasts (GJH vs non-GJH)^a^

Time point	Group	Adjusted mean (HHS)	Mean difference (GJH − non-GJH)	95% CI for mean difference		*p*^c^
				**Lower Bound**	**Upper Bound**	
preoperative	GJH	41.31^b^	− 1.83	− 6.78	3.11	0.462
	non-GJH	43.15^b^	-	-	-	-
1 month	GJH	58.65^b^	− 10.72	− 13.34	− 8.10	< 0.001
	non-GJH	69.36^b^	-	-	-	-
3 months	GJH	74.69^b^	− 6.24	− 8.67	− 3.80	< 0.001
	non-GJH	80.93^b^	-	-	-	-
6 months	GJH	81.79^b^	− 3.66	− 5.42	− 1.89	< 0.001
	non-GJH	85.44^b^	-	-	-	-
12 months	GJH	85.84^b^	− 0.82	− 2.59	0.95	0.356
	non-GJH	86.66^b^	-	-	-	-

^a^Dependent variable: Harris Hip Score (HHS)

^b^Estimated marginal means are adjusted to the covariate values specified by the model: Cobb angle = 10.081°, femoral offset (cm) = 3.3866, and osteotomy shortening distance (cm) = 1.1072

^c^*p*-values are Bonferroni-adjusted for multiple comparisons

To evaluate the robustness of these findings to the baseline imbalance in Tönnis grade, a sensitivity analysis was performed restricted to Tönnis grade 0–1 patients (GJH *n* = 21; non-GJH *n* = 26; total *n* = 47). The direction and significance pattern of all fixed effects were consistent with the primary analysis: time, GJH group, and the time × group interaction all remained significant, while the covariates remained non-significant. Detailed results are provided in Supplementary Table S1.

### Major complications and reoperations

During follow-up, one major postoperative complication was recorded, and it occurred in the GJH group. One patient experienced a prosthetic dislocation after the index procedure. Closed reduction was successful, and no recurrent dislocation occurred during follow-up. No patient underwent revision arthroplasty. Serial radiographs obtained throughout follow-up showed no evidence of femoral stem subsidence or component loosening in either group. All implants maintained stable alignment and fixation.

## Discussion

This study examined whether GJH influences functional recovery after THA for unilateral Hartofilakidis type C DDH. The central finding is that GJH was associated with a different recovery trajectory rather than an inferior short-term endpoint. In the LMM, the time × group interaction was significant, indicating that the rate of improvement differed between groups. Patients with GJH showed slower early recovery, whereas the between-group difference was not statistically significant at 12 months. This aligns with the descriptive HHS data, which showed large early separation and subsequent convergence.

Hartofilakidis type C DDH presents specific technical risks. Reduction and limb-length restoration can place the sciatic nerve at risk, and excessive acute lengthening has been linked to postoperative sciatic nerve injury in adult hip dislocation THA [18, 19]. These concerns motivated our strategy of combining THA with an exposure-oriented PFO and selective femoral shortening when needed. Recent reports of DAA-based reconstructions combined with proximal femoral osteotomy in Crowe IV/DDH emphasize improved femoral exposure and feasibility for complex reductions, with acceptable implant stability [16, 17, 20]. In addition, nerve injury remains a recognized complication in Crowe IV reconstructions using osteotomy and shortening techniques, reinforcing the need to balance reduction with neurovascular safety [21]. In our cohort, leg-length discrepancy was substantially corrected, and radiographs showed stable fixation without stem subsidence or component loosening, supporting the mechanical reliability of the reconstruction in both groups.

Evidence from hypermobility-related connective tissue disorders suggests higher rates of implant-related complications after THA, including dislocation, which underscores the importance of stability optimization in laxity-prone patients [22–24]. Although Ehlers–Danlos syndrome (EDS) is not synonymous with GJH, it provides clinically relevant context that reduced soft-tissue restraint can increase instability susceptibility. [24] In our series, only one dislocation occurred, and it was treated successfully with closed reduction. The event rate was too low to support comparative inferences. Nevertheless, our approach used a DAA in all cases, and contemporary evidence indicates that DAA has a dislocation risk comparable to the posterior approach in primary THA, while offering potential advantages in early recovery in some settings [25]. In patients with generalized laxity, the surgical team considered soft-tissue tension and stability during trial reduction and adjusted offset when necessary. This was achieved by modifying the depth of the trochanteric groove and selecting an appropriate neck option, with the aim of optimizing periarticular tension and reducing instability risk. In the present cohort, femoral offset was numerically higher in the GJH group, which is consistent with this stability-oriented strategy. Restoration of femoral offset contributes to soft-tissue tension and stability after THA, and insufficient restoration has been associated with an increased risk of dislocation [26].

In our cohort, the between-group separation was most pronounced in the early postoperative period and progressively narrowed thereafter. Model-based contrasts indicated a large adjusted deficit in the GJH group at 1 month, a moderate deficit at 3 months, and a smaller but still detectable deficit at 6 months; by 12 months, the groups converged with no statistically significant difference. To complement statistical inference, we interpreted the magnitude of the GJH effect against established benchmarks for clinical relevance. We adopted a minimal clinically important difference (MCID) of 7.9 points for the HHS [27]. Applying this threshold to the adjusted between-group differences from the LMM (Table 5), the GJH effect was both statistically significant and clinically meaningful at 1 month (adjusted difference − 10.72), borderline at 3 months (− 6.24), statistically significant but clinically negligible at 6 months (− 3.66), and absent at 12 months (− 0.82). We additionally contextualized our findings against the Patient Acceptable Symptom State (PASS) threshold reported by Galea [28]. At 3 months postoperatively, the adjusted mean HHS in the GJH group (74.69) fell just below the 3-month PASS threshold of 76 points, whereas the non-GJH group (80.93) had crossed it, indicating that GJH patients were less likely to have reached an acceptable symptom state at this early stage. Together, these analyses identify the first 1–3 postoperative months as a critical intervention window for patients with GJH, supporting targeted counseling and closer follow-up during this period to maintain rehabilitation adherence and to address early functional limitations.

Mechanistically, slower early recovery in GJH is plausible. Hypermobility spectrum disorders have been linked to impaired proprioceptive acuity and, in some patients, altered pain processing, which may affect movement confidence, pain perception, and tolerance of early rehabilitation loads [29]. Reduced passive stability may also increase reliance on neuromuscular control and peri-hip muscle performance, potentially delaying gait normalization and functional progression after technically demanding reconstructions [7, 30, 31]. Because the rehabilitation protocol applied in this study was uniform across groups and was not stratified by GJH status, the divergent recovery trajectory observed in patients with GJH is unlikely to be an artifact of differential rehabilitation intensity, and more plausibly reflects a biologically meaningful effect of generalized laxity on early functional recovery. Building on our findings and the existing literature on rehabilitation in hypermobile populations [32–34], several refinements may be considered for GJH-tailored programs after THA: (i) milestone-based rather than time-based progression, given the slower early functional gains observed in this group; (ii) emphasis on dynamic stability, proprioceptive control, and progressive abductor strengthening during the 1–3 month window, identified in our data as the period of maximal between-group divergence; (iii) structured gait retraining during the 3–6 month window, when residual functional differences may persist; (iv) mid-range joint control training to reduce dependence on end-range passive support; and (v) expectation management and psychological support, since slower early progress may itself reduce motivation and adherence. These recommendations are framed as hypothesis-generating, and prospective controlled trials are required to validate the value of GJH-stratified rehabilitation strategies.

We also explored lumbar coronal alignment because unilateral high dislocation DDH is commonly associated with pelvic obliquity, leg-length discrepancy, and compensatory lumbar curvature [35, 36]. In our baseline data, the lumbar Cobb angle was larger in the GJH group, but the Cobb angle was not independently associated with longitudinal HHS recovery in the LMM. This negative finding should be interpreted conservatively. The study may be underpowered to detect modest effects, and HHS is hip-focused and may not fully capture spine-related symptoms. Moreover, we did not reassess lumbar alignment at 1 year. Therefore, we cannot determine whether coronal compensation changed after reconstruction or whether changes differed by GJH status. The hip–spine relationship remains relevant for arthroplasty function and stability, and future work should include standardized postoperative spinopelvic reassessment and spine-specific outcomes to clarify mechanisms [37].

We limited inclusion to young adults with unilateral Hartofilakidis type C DDH to isolate the association of GJH with recovery trajectory and to avoid confounding from staged bilateral reconstructions, where contralateral pathology and inter-stage interval influence gait and patient-reported outcomes. This cohort represents a young unilateral high-dislocation DDH population in whom limb-length discrepancy and gait impairment may justify reconstruction even when radiographic osteoarthritis is mild. In our sample, the overall distribution was dominated by Tönnis grade 0–1, although the non-GJH group included a higher proportion of grade 2–3 disease. Importantly, Tönnis grade was not an independent predictor of longitudinal HHS in the multivariable LMM (*p* = 0.145), which suggests that baseline differences in radiographic osteoarthritis severity were unlikely to explain the observed divergence in early recovery trajectories. A prespecified sensitivity analysis restricted to Tönnis grade 0–1 patients (*n* = 47) reproduced the principal findings: time, GJH group, and the time × group interaction all remained significant, while the included covariates remained non-significant (Supplementary Table S1). This indicates that the baseline imbalance in Tönnis grade did not materially alter the principal conclusions.

Several limitations should be acknowledged. First, the retrospective single-center design inherently limits causal inference, and residual selection bias cannot be entirely excluded—particularly from the exclusion of two patients without a documented preoperative Beighton score. Sensitivity analyses indicated that this bias is likely minimal, but external multicenter validation is warranted. Second, the modest sample size, especially in the GJH subgroup (*n* = 21), limits statistical power for rare events such as dislocation, periprosthetic fracture, and revision; complication-related and subgroup findings should therefore be regarded as exploratory and hypothesis-generating. Unilateral Hartofilakidis type C DDH in adults aged 18–40 years is, however, an uncommon condition, and our sample size is comparable to prior single-center series. Third, baseline functional assessment relied on the HHS, and no patient-reported outcome measures were collected at baseline; broader functional dimensions could not, therefore, be characterized. Fourth, although a uniform stage-based rehabilitation protocol was applied, quantitative adherence data were not systematically collected; adherence may itself lie on the causal pathway between GJH and recovery (i.e., a potential mediator rather than a pure confounder), as patients with slower early functional gains may experience reduced motivation. Prospective studies employing structured adherence monitoring are needed. Fifth, the Beighton score is a single-dimensional instrument that captures joint range of motion only and cannot distinguish asymptomatic hypermobility from Hypermobility spectrum disorder or hypermobile EDS, nor capture multisystem manifestations of hereditary connective-tissue disorders [38]. Sixth, the MCID (7.9 points) and 3-month PASS (76 points) thresholds applied for clinical interpretation were not derived from cohorts with Hartofilakidis type C high-dislocation DDH; although these benchmarks provide a useful frame of reference, their direct applicability to our population is uncertain and may introduce interpretive bias, and prospective derivation of MCID and PASS values specific to this severe DDH subgroup is warranted. Finally, postoperative lumbar Cobb angle and lumbar lordosis were not reassessed at 1 year, so spinopelvic compensation after reconstruction could not be evaluated.

Despite these limitations, the findings support that GJH does not reduce 12-month hip function after THA for unilateral Hartofilakidis type C DDH, but it is associated with slower early recovery. These results justify explicit preoperative identification of GJH, intraoperative attention to stability-related reconstruction parameters, and an early rehabilitation strategy that emphasizes neuromuscular control and strengthening.

## Conclusion

In this retrospective cohort of young adults with unilateral Hartofilakidis type C DDH, total hip arthroplasty performed through a direct anterior approach with proximal femoral osteotomy for exposure and selective femoral shortening achieved substantial correction of leg-length discrepancy, stable radiographic fixation, and a low rate of major complications. Generalized joint hypermobility was not associated with reduced 12-month hip function. Still, it was associated with a clinically and statistically meaningful slower early recovery trajectory, with the largest functional deficit observed during the first 1–3 postoperative months, followed by progressive convergence between groups thereafter. These findings support preoperative identification of GJH, careful intraoperative stability management, and a rehabilitation program that emphasizes early neuromuscular control and peri-hip strengthening in patients with generalized laxity.

## Supplementary Information

Supplementary Material 1: Supplementary file 1. Diagnostic criteria for generalized joint hypermobility (GJH), Supplementary Table S1. Sensitivity analysis of fixed effects in linear mixed-effects models.

## Data Availability

The datasets generated and/or analysed during the current study are available from the corresponding author upon reasonable request. The datasets are not publicly available because they contain information that could compromise participant privacy and because of institutional/ethical restrictions. De-identified data can be shared following approval by the institutional review board and, where applicable, execution of a data use agreement.
