# Supplementary material for: Generalized joint hypermobility and functional recovery after THA for unilateral Hartofilakidis type C developmental dysplasia of the hip: a retrospective cohort study
Source: Arthroplasty. 2026 Jun 9;8:46. doi: 10.1186/s42836-026-00405-7 (PMC13248390; doi:10.1186/s42836-026-00405-7)
Supplement: Supplementary file 1 — Supplementary Material 1: Supplementary file 1. Diagnostic criteria for generalized joint hypermobility (GJH), Supplementary Table S1. Sensitivity analysis of fixed effects in linear mixed-effects models. [file 42836_2026_405_MOESM1_ESM.docx]

**Supplementary materials**

**Supplementary file 1. Diagnostic criteria for generalized joint hypermobility (GJH)**

Beighton examination (maximum score 9):

- Passive dorsiflexion/extension of the fifth metacarpophalangeal joint beyond 90° (examiner supports the palm and forearm on a flat surface with the elbow flexed at 90°) — 1 point for each side.
- Passive apposition of the thumb to the volar aspect of the ipsilateral forearm, with the arm extended forward and the forearm pronated — 1 point for each side.
- Passive hyperextension of the elbow more than 10°, with the arms held by the sides and the forearms supinated — 1 point for each side.
- Passive hyperextension of the knee more than 10° while standing with the knees fully extended — 1 point for each side.
- Active forward flexion of the trunk with the knees fully extended so that the palms rest flat on the floor — 1 point.

Age-specific thresholds commonly used:

1. Prepubertal children and adolescents: Beighton score ≥ 6.
2. **From puberty up to 50 years of age: Beighton score ≥ 5.**
3. Persons older than 50 years: Beighton score ≥ 4.

If the Beighton score is one point below the age- and sex-specific cutoff, two or more of the following must also be selected to meet the criterion

1. Can you now (or could you ever) place your hands flat on the floor without bending your knees?

2. Can you now (or could you ever) bend your thumb to touch your forearm?

3. As a child, did you amuse your friends by contorting your body into strange shapes, or could you do the splits?

4. As a child or teenager, did your shoulder or kneecap dislocate on more than one occasion?

5. Do you consider yourself “double-jointed”?

**Supplementary Table S1**. Sensitivity analysis of fixed effects in linear mixed-effects models

|  | **Num DF** | **Den DF** | **F** | ***p*** |
| --- | --- | --- | --- | --- |
| Intercept | 1 | 34.97 | 329.89 | <0.001 |
| Time | 4 | 79.62 | 534.16 | <0.001 |
| GJH Status | 1 | 34.80 | 7.96 | 0.008 |
| Tönnis Classification | 1 | 34.98 | 0.43 | 0.514 |
| Cobb Angle (°) | 1 | 34.98 | 0.27 | 0.610 |
| Femoral Offset (cm) | 1 | 34.98 | 0.51 | 0.482 |
| F-OSD (cm) | 1 | 34.98 | 2.03 | 0.163 |
| Time × GJH Status | 4 | 79.62 | 11.06 | <0.001 |

Dependent variable: Harris Hip Score (HHS); sensitivity analysis included only patients with Tönnis grade 0–1 (*n* = 47).
